# The future of plastic

**DOI:** 10.1038/s41467-018-04565-2

**Published:** 2018-06-05

## Abstract

Plastic, a highly useful and convenient material, is also one of the world’s greatest environmental problems, yet both industry and society are still heavily reliant on its usage. On World Environment Day, *Nature Communications* asks: will biodegradable polymers alleviate plastic’s environmental impact?

From initial conception, plastic was hailed a wondrous material. Following 80 years of innovation involving disciplines spread across industry and academia, mass production of plastic became successful and revolutionised consumerism in a post-World War II generation^1^. Plastic, although a simple synthetic polymer consisting of small molecules (monomers) linked together in a repetitive formation, is extremely versatile; with properties ranging from, resistance to corrosion, light weight, high strength, transparency, low toxicity to durability. Used by almost every industry in the world, from food packaging to space exploration, plastic is the ultimate commodity of convenience. Household names in the plastic industry include polyethylene terephthalate (PET), polyethylene (PE), polypropylene (PP), polystyrene (PS) and polyvinyl chloride (PVC).^1^

**Figure:**
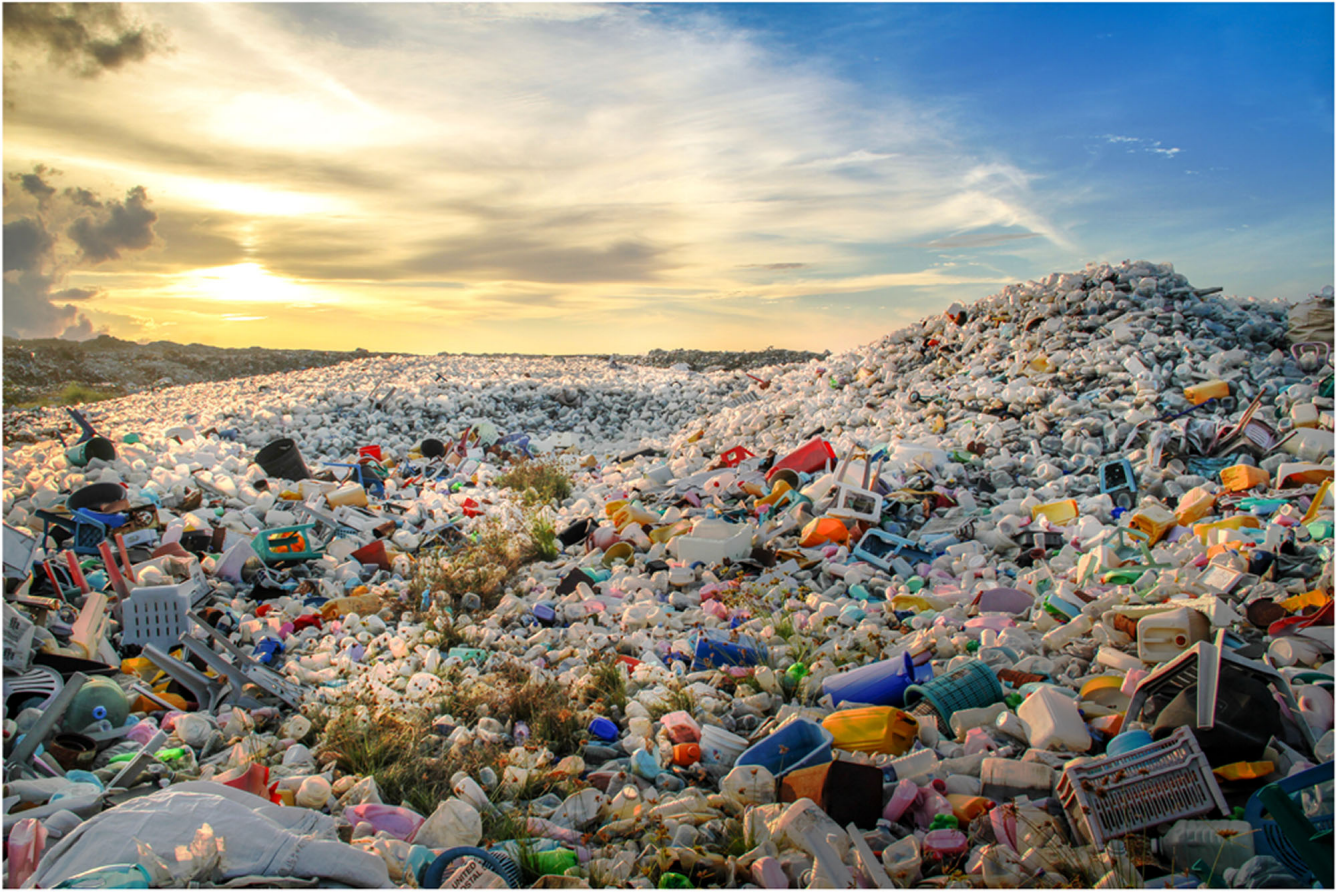
AbdulRaheemMohamed/EyeEm/Getty

“Durability, one of plastic’s greatest assets is now its curse—its robustness means that plastics stay in our environment for hundreds of years.”

Although the ease of plastic production generates cheap goods, the linear plastic economy adopted sees 90% of products used once and then discarded, thus creating a global environmental crisis. Since the plastic revolution, 6.3 billion tonnes of plastic waste has been produced worldwide^2^. We store roughly 79% of plastic waste in landfills, which results in up to 2.41 million tonnes of plastic waste entering oceans via rivers every year^3,4^. Durability, one of plastic’s greatest assets is now its curse–its robustness means that plastics stay in our environment for hundreds of years. Even when degraded, plastic never truly leaves the environment but is present as smaller pieces invisible to the naked eye (microplastics) that are choking marine life and propagating up the food chain^5^. Alongside a solution to the existing plastic waste problem, a new plastic future is also required.

Reduce, reuse and recycle have been embraced as the common approach to combat the escalating plastic waste problem. The dream is to create a circular plastic economy where products are 100% recyclable, used for as long as possible, and their waste is minimised^3,6^. Until recently this strategy has lacked success, but with an increasing number of new initiatives, support from governments and leading manufacturers committing to achievable targets, change is being accomplished^6^. For now, progress remains slow despite advances in molecular level recycling, which enables different plastics to be recycled together^7,8^. Recycling is costly, reliant on human behavioural changes and produces lower quality materials, in terms of both thermal and mechanical properties^7^. Additionally, recycling does not curb our plastic addiction; if we want to maintain our current lifestyles modification to plastic manufacture needs to go hand in hand with effective recycling.

Recent success in reducing carrier bag (PE) and drinks bottles (PET) waste in Europe suggests lifestyle adjustments are possible, but plastic is ingrained in modern society and a future free from plastic seems unlikely. Complete alteration of human behaviour is difficult to attain, as indicated by the fact that only 9% of plastic waste is recycled^3^. Therefore in addition to these three solutions to the plastic waste problem (reducing, reusing and recycling), we need a fundamental change in order to make a noticeable impact on the plastic waste seeping into our environment. A new plastic future in which biodegradable polymers replace conventional plastics could be the answer.

Biodegradable polymers can break down into smaller molecules, such as CO_2_, CH_4_ and H_2_O, by microorganisms under aerobic or anaerobic conditions. Although not always required, abiotic chemical reactions like photodegradation, oxidation and hydrolysis can also aid the degradation process^9^. There are many examples of biodegradable polymers, some are produced from plants, animals or micro-organisms, others are purely synthetic (man-made). The most commonly known synthetic biodegradable polymers are polylactide (PLA), polyglycolide (PGA), polycaprolactone (PCL), polyhydroxyalkanoates (PHA), poly(butylene succinate) (PBS) and poly(butylene adipate-*co*-terephthalate) (PBAT)^9^.

PLA is considered the most promising candidate to replace current plastics. Unlike other synthetic biodegradable polymers and even conventional plastics, which are produced from petrochemicals, PLA is formed from sustainable resources (lactic acid in corn)^9,10^. However, if such biodegradable polymers were produced on an industrial scale, competition for land with food crops may become an issue. Good mechanical strength and low toxicity have already led to PLA’s successful implementation in packaging and biomedical applications^9^. Unfortunately, PLA has one important downside–its poor thermal properties limit its applicability at high temperatures (above 60 °C)^11^.

Despite PLA’s shortcomings, interest in this material has not waned due to its faster degradation time compared to current plastics (~12 months), which is believed to prevent its accumulation in our environment if implemented on an industrial scale^12^. However, specific micro-organisms present in composting plants at slightly elevated temperatures are required for this process; if not available the degradation time can be longer. The small molecules formed during biodegradation do not impact the environment in the same way as microplastics, but there are concerns that they will add to our greenhouse gas (GHG) emissions. That said, life cycle analysis has found that less net GHG generation occurs during PLA production compared to current petroleum-based plastics^13^.

Although biodegradable polymers and in particular PLA have been the focus of much research and patents over the last decade, their production has still not reached the level of PE, PET and PP due to cost^10,11,14^. Lactic acid is not as readily available compared to the starting materials used for current plastics (e.g. ethylene for PE). Additionally, lactic acid is converted to lactide before PLA can form and this extra-step adds to the final expenditure^11,14^.

Biodegradable polymers along with reducing, reusing and recycling could impact the accumulation of plastics in the environment, but further developments are still required before PLA or other biodegradable polymers can replace existing plastics^10,15^. Cost is not the only roadblock for such materials. Governments, society and industry have learnt from past mistakes and realise that production of new materials must consider their source and end of life together with the essential criteria of production scalability and material properties. In order to successfully substitute current plastics with biodegradable polymers, we not only need industry and academia to work together but also different disciplines (chemistry, engineering, materials science, biogeochemistry and climate science) to collaborate. Similar to the current plastics we use, this process will take time and key multi-disciplinary developments will be required. We hope *Nature Communications* provides the interdisciplinary, open-access platform to disseminate this research to all relevant stake-holders. We have begun the journey towards a new plastic future involving biodegradable polymers; we need to persevere together to reach the finish line in order to protect our environment.

## References

[1] Feldman D (2008). Polymer history. Des. Monomers Polym..

[2] *The New Plastics Economy: Rethinking the Future of Plastics*. https://www.ellenmacarthurfoundation.org/publications/the-new-plastics-economy-rethinking-the-future-of-plastics (Ellen MacArthur Foundation, 2016).

[3] Geyer R, Jambeck JR, Law KL (2017). Production, use, and fate of all plastics ever made. Sci. Adv..

[4] Lebreton LCM (2017). River plastic emissions to the world’s oceans. Nat. Commun..

[5] Romera-Castillo C, Pinto M, Langer TM, Álvarez-Salgado XA, Herndl GJ (2018). Dissolved organic carbon leaching from plastics stimulates microbial activity in the ocean. Nat. Commun..

[6] *A European Strategy for Plastics in a Circular Economy*. http://eur-lex.europa.eu/legal-content/EN/TXT/?qid=1516265440535&uri=COM:2018:28:FIN (European Commission, 2018).

[7] Eagan JM (2017). Combining polyethylene and polypropylene: enhanced performance with PE/iPP multiblock polymers. Science.

[8] *PET Cradle-to-Cradle solution “..a Game Changer..”* http://www.ioniqa.com/pet-recycling/ (2018).

[9] Luckachan GE, Pillai CKS (2011). Biodegradable polymers–a review on recent trends and emerging perspectives. J. Polym. Environ..

[10] Elvers D, Song CH, Steinbüchel A, Leker J (2016). Technology trends in biodegradable polymers: evidence from patent analysis. Polym. Rev..

[11] Jamshidian M, Tehrany EA, Imran M, Jacquot M, Desobry S (2010). Poly-lactic acid: production, applications, nanocomposites, and release studies. Compr. Rev. Food Sci. Food Saf..

[12] Song JH, Murphy RJ, Narayan R, Davies GBH (2009). Biodegradable and compostable alternatives to conventional plastics. Philos. Trans. R. Soc. Lond. B Biol. Sci..

[13] Cosate de Andrade MF, Souza PMS, Cavalett O, Morales AR (2016). Life cycle assessment of Poly(Lactic acid) (pla): comparison between chemical recycling, mechanical recycling and composting. J. Polym. Environ..

[14] Dusselier M, Van Wouwe P, Dewaele A, Jacobs PA, Sels BF (2015). Shape-selective zeolite catalysis for bioplastics production. Science.

[15] Shen L, Worrell E, Patel M (2010). Present and future development in plastics from biomass. Biofuels, Bioprod. Bioref..

